# Free Sugar Intake and Periodontal Diseases: A Systematic Review

**DOI:** 10.3390/nu14214444

**Published:** 2022-10-22

**Authors:** Taro Kusama, Noriko Nakazawa, Kenji Takeuchi, Sakura Kiuchi, Ken Osaka

**Affiliations:** 1 Division for Regional Community Development, Liaison Center for Innovative Dentistry, Tohoku University Graduate School of Dentistry, Sendai 980-8575, Japan; 2 Department of International and Community Oral Health, Tohoku University Graduate School of Dentistry, Sendai 980-8575, Japan; 3 Frontier Research Institute for Interdisciplinary Sciences, Tohoku University, Sendai 980-8578, Japan

**Keywords:** oral health, periodontitis, gingivitis, diet, sugar-sweetened beverages, confection, snack, non-communicable disease

## Abstract

High free sugar intake is associated with an increased risk of various non-communicable diseases. We aimed to systematically review articles investigating the association between free sugar intake and periodontal diseases. This systematic review was conducted according to PRISMA guidelines and was registered in the PROSPERO database (CRD42022337828). We obtained articles from PubMed, Web of Science, and Scopus in April 2022. The study selection was performed according to predefined eligibility criteria based on the following PECOS: (P) general population, (E/C) free-sugar-containing food/beverage intake, (O) clinically measured periodontal diseases, and (S) observational study and clinical trial. Of the 839 screened records, 13 studies were included in the review. Most studies (n = 12) had a cross-sectional design. The age groups in the included studies were children/adolescents (n = 5) and adults (n = 8). Among the included studies, 11 reported a significant association between the frequent intake of free-sugar-containing food or beverages and a higher prevalence or incidence of periodontal diseases. The quality of most of the included studies was scored “fair” based on the Newcastle–Ottawa Quality Assessment Scale. Although the majority of the included studies reported a significant positive association between high free sugar intake and periodontal diseases, the evidence is considered to be limited due to the study designs.

## 1. Introduction

Periodontal diseases, including periodontitis and gingivitis, are among the most prevalent diseases worldwide, and their prevalence increases with age [1,2]. Besides their clinical symptoms and economic burden [3], periodontal diseases subsequently increase the risk of various systemic diseases, including diabetes [4], respiratory diseases [5], dementia [6], cardiovascular diseases [7], and mortality [8]. Therefore, the prevention of periodontal disease is an important global health issue. Although previous studies have shown several risk factors for periodontal disease [1], an effective population approach for periodontal disease prevention is limited.

Sugar intake restriction is considered an effective public health countermeasure to prevent various health problems [9]. In recent decades, high free sugar intake has been recognized as a common risk factor for various non-communicable diseases, including dental caries [10], obesity [11], diabetes [12], cardiovascular diseases [13], and cancer [14]. Free sugars are monosaccharides (i.e., glucose, fructose, and galactose) and disaccharides (i.e., sucrose, lactose, maltose, and trehalose) added to foods or beverages [15].

Although sugar control has received much attention in relation to the prevention of dental caries [10], it has rarely been mentioned in relation to the prevention of periodontal diseases. However, a few previous studies have reported an association between frequent free sugar intake and the prevalence of periodontal diseases [16,17]. A recent review referring to the connection between dental caries and periodontal diseases also mentioned several studies reporting an association between free sugar intake and periodontal diseases [18]. A previous systematic review revealed that poor dietary intake (e.g., less consumption of vegetables or fruits) was positively associated with periodontal diseases [19]; however, to the best of our knowledge, no systematic review has reported an association between free sugar intake and periodontal diseases. Our research question was, “Is there any association between the higher intake of free-sugar-containing food or beverage and periodontal diseases?” We aimed to systematically review existing articles investigating the association between higher free sugar intake and the risk of periodontal diseases and to synthesize these findings. In addition, we only targeted studies that employed clinical measures to evaluate periodontal disease in order to reduce information bias.

## 2. Methods

### 2.1. Registration and Protocol

We followed the Preferred Reporting Items for Systematic Reviews and Meta-Analysis (PRISMA) guidelines to conduct this systematic review [20]. This systematic review was registered with PROSPERO (CRD42022337828).

### 2.2. Search Strategy

Our research question can be translated to PECOS as follows: (P) general population; (E) higher intake of free-sugar-containing food or beverages; (C) no or lower intake of free- sugar-containing food or beverages; (O) periodontal diseases (gingivitis/periodontitis/peri-implantitis), which were measured clinically; (S) observational study (except for ecological study) and clinical trial. We performed a literature search using the PubMed, Web of Science, and Scopus databases. We included articles published from 1 January 2000 to 26 April 2022. We selected relevant keywords to find articles based on PECOS. The Boolean search strings were (gingivitis OR “periodontal disease” OR periodontitis) AND (“fermentable carbohydrate” OR “carbonated beverage” OR sugar OR snack OR confectionery OR juice OR soda) AND (“cross-sectional” OR cohort OR longitudinal OR “case-control” OR “case-cohort” OR “randomized controlled” OR “clinical trial”). In addition, we also performed a manual search through the reference lists of the original articles and reviews to identify further relevant studies. The search was restricted to studies that investigated human subjects and that were published in English.

### 2.3. Study Selection

**Figure 1:**
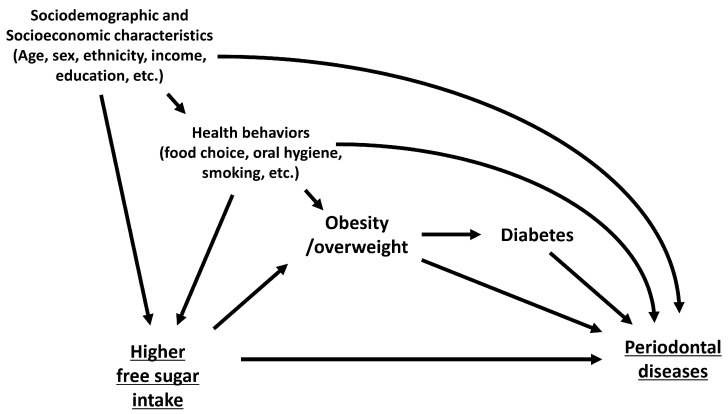
Causal diagram of the association between higher free sugar consumption and periodontal diseases.

We selected studies that met the following eligibility criteria: (1) studies that targeted the general population and were not restricted to those with certain diseases or disabilities; (2) studies that evaluated the frequency or amount of free sugar intake from any food or beverage, including snacks, candy, and sugar-sweetened beverages (SSB); (3) studies that employed clinical indicators or indexes of periodontal diseases as the outcome, including probing pocket depth (PPD), clinical attachment level (CAL), and the bleeding of pockets (BOP); (4) observational studies and clinical trials that evaluated individual-level associations, including cohort, cross-sectional, and case–control studies, as well as randomized clinical trials; and (4) studies that considered relevant covariates, especially for confounders, and that estimated adjusted effect size (odds ratio, relative risk, prevalence ratio, etc.). We hypothesized that the possible confounders in the relationship between high free sugar intake and periodontal diseases are sociodemographic factors (e.g., sex, age, and ethnicity), socioeconomic factors (e.g., income and education), and health behavior (e.g., smoking, food choice, oral hygiene, and physical activity) based on previous studies (Figure 1) [1,4,11,12,21,22,23]. Obesity/overweight and diabetes are considered intermediate variables in the relationship between frequent sugar intake and periodontal diseases. In addition, we excluded studies that met the following criteria: (1) the intake of free sugar was evaluated with other food groups that do not usually add free sugars; (2) self-reported symptoms of periodontal diseases were employed as the outcome; and (3) an ecological or a secondary analysis was performed.

For the study selection process, two investigators (T.K. and N.N.) independently conducted study screening to identify eligible studies. Initially, we selected eligible studies by screening only the titles and abstracts. We included studies that both investigators judged to be eligible for the next screening step. In the next step, we obtained the full-text articles of the studies that were judged as being eligible in the initial step. We read the entire text and checked whether each study met the eligibility criteria of the review as detailed above. If there was a discrepancy in the judgment of each study between the two investigators, we discussed whether the study was eligible.

### 2.4. Data Extraction and Quality Assessment

An investigator (T.K.) reviewed each eligible study and extracted the following data: first author’s name, the year of publication, study design, geographical location, study’s targeted population, the number of participants, sex and age distribution in the study population, clinical measurements that evaluated periodontal diseases, the prevalence or incidence of periodontal diseases, the source of free sugar, the categories of exposure, included covariates, statistical methods, estimates of association, funding source, and conflicts of interest.

The quality of each study was assessed using the Newcastle–Ottawa Quality Assessment Scale (NOS) [24]. The NOS was originally created for cohort studies and case–control studies; therefore, we also employed a modified version of the NOS, which was fitted for cross-sectional studies (Supplementary Text S1a,b) [25]. The NOS score ranges from 0 to 8, with higher scores indicating higher study quality.

In this review, we found large heterogeneity in the outcome and exposure variables used in each included study; therefore, we could not conduct a meta-analysis to synthesize the results of the included studies.

## 3. Results

### 3.1. Literature Search

**Figure 2:**
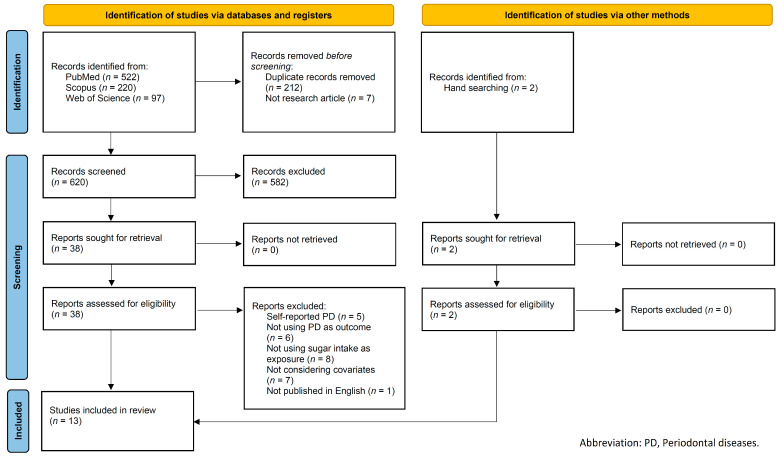
Flow diagram of literature selection.

We initially identified 839 articles from the electronic databases, and 212 and 7 articles were excluded due to duplication and publication type, respectively. Of the 620 articles, two investigators (T.K. and N.N.) independently screened each article by title and abstract. The proportion of agreement on inclusion and exclusion between the two investigators was 97.4% (kappa = 0.78). After discussing the discrepancy in judgment between the two investigators, 38 articles were selected for full-text screening. Subsequently, the two investigators independently evaluated the eligibility of each article by full-text reading, and they identified 11 eligible articles based on our inclusion and exclusion criteria. In addition, two articles were identified through manual searches. Ultimately, 13 articles were included in this review (Figure 2).

### 3.2. Study Characteristics

The descriptive characteristics of the included 13 studies are presented in Table 1. The included studies were conducted in various countries: five in Asia [16,26,27,28,29], three in America [17,30,31], two in the Middle East [32,33], two in Europe [34,35], and one in Africa [36]. Most studies had a cross-sectional design [16,17,25,26,27,28,30,31,32,33,34,35], and only one study had a cohort design [29]. The number of participants in each study ranged from 169 to 10,022. The included studies covered almost all age groups; five studies targeted schoolchildren or adolescents [26,28,32,33,36] and eight targeted adults [16,17,27,29,30,31,34,35], while Yoshihara et al. only targeted older adults aged 70 years [29]. For the eligibility of participants, one study only targeted pregnant women [30].

The detailed estimates of the association between free sugar intake and periodontal diseases are presented in Supplementary Table S1. Among the 13 included studies, 11 indicated a significant association between the frequent intake of free-sugar-containing food or beverages and a higher prevalence or incidence of periodontal diseases. Although only seven studies included sufficient confounders as covariates, including sociodemographic and socioeconomic factors, and health behaviors (Table 1), most of these studies reported significant associations between the frequent intake of free-sugar-containing food or beverages and the presence of periodontal diseases [16,17,26,27,28,31], with the exception of one study [32]. In addition, several studies included variables related to obesity or diabetes as covariates [16,17,27], which are considered to be intermediate variables in the mechanism between higher free sugar consumption and the onset of periodontal diseases (Figure 1); however, all of these studies also indicated significant associations.

The funding sources and conflicts of interest of each study are presented in Supplementary Table S2. No study stated that their studies were supported by the food industry or other industries, or companies that make a profit from sugar-related products. However, several studies did not clearly state their funding sources [16,33,36]. In addition, no study reported any conflicts of interest. However, a few studies did not clearly state the conflicts of interest in their manuscript [16,29,33,36].

### 3.3. Measurements of Periodontal Diseases

Various clinical measurements of periodontal diseases were used in the included studies. All clinical measurements were widely used to diagnose or screen for periodontal diseases. Studies that targeted school children or adolescents employed BOP or the Löe and Silness’s gingival index (GI) with a cut-off of 2 points [26,32,33,36], which mainly evaluates the presence of gingivitis. The PMA index was also used in one study targeting schoolchildren [28]. In contrast, studies targeting adults employed PPD, CAL, and their combination with BOP, or the community probing index (CPI) with a cut-off of 3 points. Most of the studies employed a cut-off of PPD ≥4 mm, which is equivalent to a CPI of ≥3 points [16,27,30,31,35]; however, one study employed a cut-off of PPD ≥3 mm with the combination of BOP [17]. We could not find any clear difference between gingivitis- and periodontitis-related clinical measures in the association between frequent free sugar intake and the presence of periodontal diseases.

### 3.4. Measurements of Free Sugar Intake

For the measurement of free sugar intake, five studies only evaluated the intake of SSB [16,26,27,30,32], while one study only evaluated the intake of free-sugar-added food [29]. The frequency of the intake of any sugary food or drink as a combined variable was employed in five studies [17,31,33,34,35], and two studies simultaneously evaluated the intake of sugary food and drinks in the same model [28,36]. Most of the studies evaluated the frequency of sugary food and/or beverages in a certain period (e.g., day, week, or month); however, one study evaluated the amount of added sugar intake using the food frequency questionnaire and estimated daily free sugar intake as a percentage of total energy intake [31]. Several studies classified the frequency of free sugar intake into multiple categories, and a dose–response relationship between a more frequent free sugar intake and a higher prevalence of periodontal diseases was observed [16,17,27,30,36]. Significant associations were observed in studies employing only SSB, sugary food, or their combination as one explanatory variable. Two studies used SSB and sugary food as separate variables and simultaneously included them in their statistical models: one study suggested a significant association with only SSB but not with sugary food [28], whereas another study suggested a significant association with only sugary food but not with SSB [36].

### 3.5. Quality Assessment

We evaluated the quality of the included studies using the NOS (Table 2). Based on the NOS, five studies scored seven or eight stars (max eight stars) [16,17,26,27,31]. In particular, the studies conducted by Lula et al. [17] and Song et al. [16] used data from nationally representative health surveys (National Health and Nutrition Examination Survey (NHANES) and Korea National Health and Nutrition Examination Survey (KNHANES), respectively) and considered the sampling weight in their analyses; therefore, they satisfied all the NOS assessment criteria.

## 4. Discussion

### 4.1. Summary of Main Findings

We performed a comprehensive systematic review to evaluate the association between higher free sugar intake and the risk of periodontal diseases, and we synthesized the findings. We identified and reviewed 13 eligible studies. Although the quality of each study was relatively high, most studies had cross-sectional designs. The majority of the studies reported a significant association between higher free sugar intake and the prevalence of periodontal diseases; therefore, the quality of the evidence of the cross-sectional association between high free sugar intake and the prevalence of periodontal diseases is relatively high. These findings were reported regardless of the source of free sugar (food or beverages) or the classification of periodontal diseases (gingivitis or periodontitis). However, the evidence of the temporal association between higher free sugar intake and the onset of periodontal diseases is limited due to the nature of the included studies.

### 4.2. Comparison with Previous Studies and Possible Explanations

Previous studies have revealed that higher free sugar intake leads to obesity and diabetes [11,12], which are risk factors for periodontal diseases [4,22]. Therefore, the findings of our review are partially supported by those of previous studies. The mechanism can be illustrated as follows: high free sugar intake leads to obesity and diabetes, and prolonged obesity and diabetes increase the risk of periodontal disease. However, several of the included studies reported a significant association between high free sugar intake and periodontal diseases among adults, even after adjusting for body mass index, diabetes, hyperglycemia, or metabolic syndrome [16,17,27].

The included studies mainly suggested the mechanism by which high sugar intake increases systemic inflammation, leading to the onset of periodontal diseases. The intake of free-sugar-added foods or beverages leads to a hyperglycemic state [37], and a prolonged hyperglycemic state increases systemic inflammation and the risk of periodontal diseases [38]. Although several studies have considered diabetes or hyperglycemia as the covariate, there is a possibility that the influence of hyperglycemia on periodontal diseases may exist even under the clinical cut-off level of hyperglycemia, which is known as pre-diabetes [38,39]. Free-sugar-supplemented foods or beverages also contain high levels of fructose in the form of sucrose or corn syrup, which increases the risk of metabolic syndrome [40]. A previous cohort study also suggested that patients with metabolic syndrome are at higher risk of periodontal disease [41]. Therefore, the subclinical or preclinical status of obesity or diabetes induced by high free sugar intake can also lead to periodontal diseases.

A previous study reported that a diet without any added free sugar reduced BOP and PPD, although subgingival bacterial counts increased, except for species commonly associated with periodontitis [42]. Therefore, the intake of added free sugar may affect not only metabolic homeostasis but also oral microbiome diversity, leading to periodontal diseases. A previous study also suggested that a diet high in sucrose is associated with a reduced diversity of the subgingival microbiome, which is a risk indicator of periodontitis [43]. In this review, we included a study targeting children and adolescents, which reported a significant association between high free sugar intake and the presence of gingivitis among these age groups [26,28,32,33,36]. Gingivitis is mainly caused by plaque accumulation or poor dental hygiene [44]. It is also possible that the change in oral microbiome diversity due to high free sugar intake affects gingival and periodontal health.

The mechanism underlying the association between high free sugar intake and onset of periodontal disease remains unclear. Future research may need to elucidate the mechanism between free sugar intake and periodontal diseases from both metabolic and microbial perspectives.

### 4.3. Strength and Limitation of Included Study

The strengths of the included studies were as follows: overall, the majority of the included studies were scored as being relatively high quality based on the NOS assessment criteria. This was due to our inclusion criteria requiring that the study employed clinical measurements of periodontal diseases and evaluated the association by considering the related covariates. The clinical measurements employed to evaluate periodontal diseases in the included studies were also based on validated or widely used diagnostic criteria for periodontitis or gingivitis [44,45]. In addition, most of the included studies conducted random sampling of the targeted population with a high response rate, which contributed to the representativeness of the participants. Furthermore, several studies included a relatively large sample size (n ≥ 2000) [16,17,26,27,31], and this also reduced the random error of the estimates.

In contrast, we also identified the limitations of the included studies as follows: First, most of the studies had a cross-sectional design, which made it difficult to determine the temporal association between high free sugar intake and the onset of periodontal diseases [46]. Further investigation employing a cohort design is required for a more robust causal inference. Second, the confounders included in the statistical models were unclear, inappropriate, or insufficient for causal inference. We initially illustrated the hypothesized causal diagram (Figure 1) and identified sociodemographic and socioeconomic factors, and health behaviors as confounders, as well as diabetes and obesity as mediators based on previous studies [1,4,11,12,21,22,23]. However, six studies included an insufficient number of confounders [29,30,33,34,35,36]. These studies failed to eliminate confounding factors in the association. In addition, several studies included confounders and mediators of association in their statistical models [16,17,27]. This would have blocked the pathway between high free sugar intake and periodontal diseases and underestimated the association [47]. Future studies should select covariates based on confounder selection criteria by drawing a causal diagram based on domain knowledge for appropriate causal inference [48].

### 4.4. Strengths and Limitations in the Review Process

This systematic review has several strengths. First, we only included studies employing clinical measurements of periodontal diseases, which contributed to reducing the information bias of the study results. Second, we included studies that considered covariates in their statistical analyses. We initially constructed a hypothesized causal diagram of the association between free sugar intake and periodontal disease, with related factors, including confounders and mediators. Therefore, we qualitatively evaluated the possibility of confounding in each of the included studies. Third, we used multiple electronic databases to obtain pieces of literature, and each searched article was independently screened by two investigators. This review process ensured the comprehensiveness of this systematic review [49]. However, this review had several limitations. We did not include pieces of gray literature or articles published in a non-English language. This may have induced selection bias in our results. Although we additionally searched for gray literature from ClinicalTrials.gov and the World Health Organization’s International Clinical Trials Registry Platform (ICTRP), we could not find additional studies related to our review question. Therefore, the probability that gray literature affects our results is low.

### 4.5. Generalizability of the Result

The included studies were conducted in various regions and covered a wide range of age groups; therefore, the association between high free sugar intake and periodontal diseases would be observed in populations other than those who participated in the included studies, including gingivitis in children and adolescents and periodontitis in adults. However, we only included one study that targeted older adults, and further studies evaluating older adults may be required to justify the abovementioned association in this age group. Furthermore, our review originally searched for studies that did not specify those with specific diseases or disabilities; therefore, the generalizability of the present result to patients with specific diseases or disabilities remains unknown. Although the detailed mechanism remains unclear, the connection between free sugar intake and periodontal diseases is supported by previous findings on metabolic and microbial aspects.

### 4.6. Implications from the Present Study

As an implication of the present study, future studies with a longitudinal design that consider appropriate confounders in the association between high free sugar intake and the onset of periodontal diseases based on a causal diagram constructed from previous findings are required. Most of the studies included in our review used cross-sectional designs, and the procedures for covariate selection were unclear; therefore, further investigation based on an appropriate causal inference framework is essential to justify the relationship between high free sugar intake and the onset of periodontal diseases [50].

From a public health viewpoint, it has been pointed out that increased free sugar intake is a risk factor for dental caries and obesity, and they subsequently increase the risk of various types of non-communicable diseases, including diabetes, cardiovascular diseases, and non-alcohol fatty liver [51]. Although the present review does not provide obvious evidence that high free sugar intake increases the risk of periodontal diseases, we believe that limiting free sugar intake would contribute to preventing periodontal diseases because there is evidence that obesity and diabetes are risk factors for periodontal diseases [4,22]. The WHO has already proposed guidelines for free sugar intake to prevent non-communicable diseases [51]. One study included in our review reported that those who complied with the WHO’s guideline (i.e., <10% sugar intake of total energy) were at lower risk of periodontal diseases [31]. Therefore, we need to limit individual free sugar intake from upstream interventions (e.g., policy, taxation, regulation, and reformulation) through midstream interventions (e.g., food environment) to downstream interventions (e.g., food labeling and education) [52]. A holistic approach to reducing free sugar intake would contribute to preventing various types of non-communicable diseases, including dental caries, obesity, diabetes, and cardiovascular diseases, as well as periodontal diseases.

## 5. Conclusion

This is the first systematic review to evaluate existing articles investigating the association between high free sugar intake and periodontal disease and to assess their methodological quality. Most of the included studies reported a significant positive association between frequent free sugar intake from foods or beverages and periodontal diseases, and the overall quality of each study was relatively fair. However, most of the included studies had a cross-sectional design; therefore, evidence of the relationship between high free sugar intake and periodontal disease remains limited. Further investigations employing a longitudinal design with appropriate statistical methods to eliminate bias and confounding are required to confirm this relationship.

## Figures and Tables

**Table 1:** Descriptive characteristics of included studies.

ID	First Author (Year)	Country	Targeted Population	Participants (*n*)	Age at Baseline	Sex (Male%)	Clinical Measurement of PD	Prevalence /Incidence of PD	Source of Free Sugar	Effect Significance ^a^	Included Covariates
Cross-Sectional Study											
1	Chen, et al. [26] (2020)	China	Adolescents without serious health problems	4525	12 y	48.6%	BOP	46.6%	Sugar-containing soft drinks/soda/milk/yogurt/tea/coffee/water	*	Region, family size, father’s educational level, mother’s educational level, brushing frequency, dental floss use, dental visit
2	El Tantawi, et al. [32] (2018)	Saudi Arabia	Male students	685	13–15 y	100.0%	GI (≥2)	14.8%	Daily use of sugary drinks	-	Mother’s and father’s education, type of residence, number of households, plaque index score, brushing frequency, smoking status
3	Fann, et al. [27] (2016)	Taiwan	General population	10,022	35–44 y	37.8%	CPI (≥3)	26.7%	Soft drinks, including carbonated beverages, cola, milk tea, and juice, or asparagus juice	*	Age, sex, educational level, cigarette smoking, regular teeth brushing, BMI, hyperglycemia, WBC, intake of fruits
							LA (≥1 mm)	41.3%		*	
4	Jagahashi, et al. [33] (2012)	Syrian Arab Republic	Students	504	6–12 y	52.2%	GI (≥2)	48.8%	Sugar-containing food and beverages (chocolate, jam, cakes, biscuits, muffins)	*	Oral hygiene, tooth brushing
5	Kyaw, et al. [28] (2020)	Myanmar	Students	537	Mean: 10.6 y (1SD = 0.7)	46.7%	PMA index	Mean: 16.2 (1SD = 5.4)	Sweet snacks	-	Sex, parents’ occupations, tooth-brushing frequency, mouth-rinsing habits, dental visits, OHI-S score, bacteria level
									Sweet drinks	*	
6	Lula, et al. [17] (2014)	US	General population	2437	18–25 y	65.0%	PPD ≥ 3 mm and BOP	18.8%	Added sugar intake (food and beverages), including cakes, cookies, brownies, ice cream, ice milk, milkshakes, chocolate candy, fudge, Hi-C, Tang, Kool-Aid, cola, and soda	*	Sex, age, race/ethnicity, education, poverty–income ratio, self-reported diabetes, serum cotinine concentration, refined starchy food intake, BMI
7	Menezes, et al. [30] (2019)	Brazil	Pregnant women in 22nd to 25th week of pregnancy	1185	N/A	0.0%	PPD ≥ 4 mm and BOP	12.3%	Soft drinks	*	Maternal age, family income, pre-pregnancy obesity, diastolic blood pressure
							CAL ≥ 4 mm	16.6%	Soft drinks	-	
8	Moreira, et al. [31] (2021)	Brazil	General population	2515	18–19 y	47.2%	BOP, PPD ≥ 4mm, CAL ≥ 4mm	20.8%	Added sugar intake was estimated as the percentage of daily calories from added sugar present in beverages, such as soft drinks, fruit-flavored juice, chocolate drinks, and energy drinks, and a wide range of food groups, such as dairy products, bread, cookies, breakfast cereals, desserts, chocolate, mayonnaise, salty snacks, and cold cuts	*	Household income, adolescent educational level, sex, smoking status, alcohol use
9	Simon, et al. [36] (2003)	Ethiopia	Students in public and private schools	1736	12–18 y	44.2%	BOP and calculus	53.4%	Sweetened drinks (e.g., milk, tea, and soft drinks),	-	Staple food, teeth cleaning
									Sweets (e.g., chocolate, cakes, candy, cookies, and ice cream)	*	
10	Song, et al. [16] (2016)	South Korea	General population	5517	19–39 y	47.5%	CPI ≥ 3	12.4%	Carbonated beverages	*	Age, sex, BMI, smoking status, drinking habits, exercise, metabolic syndrome, frequency of tooth brushing, use of secondary oral products, dental checkup, consumption of coffee
11	Vilarrasa, et al. [34] (2021)	Spain	Patients with dental implants	169	Mean: 54.5 y (1SD = 11.7)	51.5%	PPD, BOP, suppuration, radiographic bone level	56.2% (Peri-implant mucositis)	Regular sugar consumption	-	Sex, oral dryness, history of periodontitis and SPT compliance, no. of caries
								22.5% (Peri-implantitis)	Regular sugar consumption	*	
12	Vitosyte, et al. [35] (2022)	Lithuania	General population	453	35–74	45.3%	Number of teeth with PPD ≥ 4 mm)	Mean: 5.9 (1SD = 5.3)	Frequency of eating or drinking any of following food/drink: cakes, sweet buns/breads, jam, honey, sweets, candies, soft drinks, tea with sugar, coffee with sugar	-	Smoking frequency, alcohol use, dental visit, use of fluoride toothpaste, tooth-brushing frequency
Cohort study											
13	Yoshihara, et al. [29] (2009)	Japan	Independent older adults	261	70 y	44.8%	No. of teeth with periodontal event (≥ 3 mm deeper PPD from baseline)	N/A	Cereals, nuts and seeds, sugar and sweeteners, confectioneries	*	Dark green and yellow vegetable intake, alcohol (g/kg), number of remaining teeth at baseline

Note: ^a^ “*” indicates a significant association between free sugar intake and periodontal disease (*p* < 0.05), and “–“ indicates a non-significant association. Abbreviations: PD, periodontal disease; BOP, bleeding on probing; GI, Löe and Sillness gingival index; CPI, community periodontal index; LA, loss of attachment; PPD, probing pocket depth; CAL, clinical attachment level; BMI, body mass index; WBC, white blood cell; OHI-S, simplified oral hygiene index.

**Table 2:** Quality assessment of included articles using the Newcastle–Ottawa scale.

Cross-Sectional Study	Chen, et al. [26] (2020)	El Tantawi, et al. [32] (2018)	Fann, et al. [27] (2016)	Jagahashi, et al. [33] (2012)	Kyaw, et al. [28] (2020)	Lula, et al. [17] (2014)	Menezes, et al. [30] (2019)	Moreira, et al. [31] (2021)	Simon, et al. [36] (2003)	Song, et al. [16] (2016)	Vilarrasa, et al. [34] (2021)	Vitosyte, et al. [35] (2022)	Cohort study	Yoshihara, et al. [29] (2009)
Newcastle–Ottawa scale assessment criteria													Newcastle–Ottawa scale assessment criteria	
Selection (Max 4 stars)													Selection (Max 4 stars)	
1. Representativeness of the sample	*	*	*	*	*	*	*	*	*	*	-	*	1. Representativeness of the exposed cohort	*
2. Sample size	*	-	*	*	*	*	*	*	*	*	*	*	2. Selection of the non-exposed cohort	*
3. Ascertainment of exposure	*	*	*	*	-	*	*	*	*	*	*	*	3. Ascertainment of exposure	*
4. Non-respondents	-	-	-	-	-	*	-	-	-	*	-	-	4. Demonstration that outcome of interest was not present at start of study	*
Comparability (Max 2 stars)													Comparability (Max 1 stars)	
1. Comparability of study results	**	**	**	-	**	**	*	**	-	**	*	-	1. Comparability of cohort	-
Outcome (Max 2 stars)													Outcome (Max 3 stars)	
1. Assessment of outcome	*	*	*	*	*	*	*	*	*	*	*	*	1. Assessment of outcome	*
2. Statistical test	*	*	*	*	*	*	*	*	*	*	*	*	2. Was follow-up sufficient	*
													3. Adequacy of follow-up	-
Total (Max 8 stars)	7	6	7	5	6	8	6	7	5	8	5	5	Total (Max 8 stars)	6

Note: Each * indicates one star.

## Data Availability

Data are available upon reasonable request.

## Supplementary Materials

The following supporting information can be downloaded at: https://www.mdpi.com/article/10.3390/nu14214444/s1, Supplementary Text S1: (a) Adapted Newcastle–Ottawa Scale for Cross-Sectional Studies; (b): Newcastle–Ottawa Scale for Cohort Studies.; Supplementary Table S1. Results of the included studies.; Supplementary Table S2. Funding sources and conflicts of interest of the included studies.
