# Supplementary material for: Free Sugar Intake and Periodontal Diseases: A Systematic Review
Source: Nutrients. 2022 Oct 22;14(21):4444. doi: 10.3390/nu14214444 (PMC9656760; doi:10.3390/nu14214444)
Supplement: Supplementary file 1 [file nutrients-14-04444-s001.zip › nutrients-1952628-supplementary.pdf]

Title: Free Sugar Intake and Periodontal Diseases: A Systematic Review

Authors: Taro Kusama, Noriko Nakazawa, Kenji Takeuchi, Sakura Kiuchi, Ken Osaka

- **Supplementary Text S1a: Adapted Newcastle-Ottawa Scale for Cross-Sectional Studies.**
- **Supplementary Text S1b: Newcastle-Ottawa Scale for Cohort studies.**
- **Supplementary Table S1. Result table of the included studies.**
- **Supplementary Table S2. Funding source and conflicts of interests of the included studies.**

Supplementary Text S1a: Adapted Newcastle-Ottawa Scale for Cross-Sectional Studies.

Selection: (Maximum of 4 stars)

1. **Representativeness of the sample:**

a) Truly representative of the average in the target population. **(all subjects or random sampling)** *
b) Somewhat representative of the average in the target population. **(non-random sampling)** *
c) Selected group of users.
d) No description of the sampling strategy.

2. **Sample size:**

a) Justified and satisfactory. *
b) Not justified.

3. **Ascertainment of exposure:**

a) Validated measurement tool. *
b) Non-validated measurement tool, but the tool is available or described. *
c) No description of the measurement tool.

4. **Non-respondents:**

a) Comparability between respondents and non-respondents characteristics is established, and the response rate is satisfactory. *
b) The response rate is unsatisfactory, or the comparability between respondents and non-respondents is unsatisfactory.
c) No description of the response rate or the characteristics of the responders and the non-responders.

Comparability: **(Maximum of 2 stars)**

1. **The subjects in different outcome groups are comparable, based on the study design or analysis. Confounding factors are controlled:**

a) The study controls for the most important factor (sex and age). *
b) The study controls for at least one additional factor on the following list. *
sociodemographic factors (sex, age, ethnicity, etc.), socioeconomic factors (income, education, etc.) and health behaviours (smoking, diet, oral hygiene, physical activity, etc.)

Outcome: (Maximum of 2 stars)

1. **Assessment of outcome:**

a) Independent blind assessment. *
b) Record linkage. *
c) Self-report. *
d) No description.

2. **Statistical test:**

a) The statistical test used to analyze the data is clearly described and appropriate, and the measurement of the association is presented, including confidence intervals or probability level (p-value). *
b) The statistical test is not appropriate, not described, or incomplete.

Supplementary Text S1b: Newcastle-Ottawa Scale for Cohort Studies.

Selection

1) Representativeness of the exposed cohort
   a) truly representative of the average _______________ (describe) in the community *
   b) somewhat representative of the average ______________ in the community *
   c) selected group of users e.g. nurses, volunteers
   d) no description of the derivation of the cohort

2) Selection of the non exposed cohort
   a) drawn from the same community as the exposed cohort *
   b) drawn from a different source
   c) no description of the derivation of the non exposed cohort

3) Ascertainment of exposure
   a) secure record (e.g. surgical records) *
   b) structured interview *
   c) written self report
   d) no description

4) Demonstration that outcome of interest was not present at start of study
   a) yes *
   b) no

Comparability

1) Comparability of cohorts on the basis of the design or analysis
   a) study controls for age and sex (select the most important factor)
   b) study controls for confounding factors as below *
   [sociodemographic factors (sex, age, ethnicity, etc.), socioeconomic factors (income, education, etc.) and health behaviours (smoking, diet, oral hygiene, physical activity, etc.)]

Outcome

1) Assessment of outcome
   a) independent blind assessment *
   b) record linkage *
   c) self report
   d) no description

2) Was follow-up long enough for outcomes to occur
   a) yes (select an adequate follow up period for outcome of interest) *
   b) no

3) Adequacy of follow up of cohorts
   a) complete follow up - all subjects accounted for *
   b) subjects lost to follow up unlikely to introduce bias - small number lost - > 80% (select an adequate %) follow up, or description provided of those lost) *
   c) follow up rate < 80% (select an adequate %) and no description of those lost
   d) no statement

Supplementary Table S1. Result table of the included studies.

ID	First author (publication year)	Targeted population	Participant (n)	Age at baseline	clinical measurement of PD	Source of free sugar	categories of exposure	Effect indicator	the estimates of association (95%CI)	statistical methods	Included covariates	Effect significance [a]
**Cross-sectional study**												
1	Chen, et al. [26] (2020)	Adolescents without serious health problem	4,525	12y	BOP	Sugar-containing soft drink/soda/ milk/yogurt /tea/coffee/water	≤1/>1 time per week	OR	1.0 (Ref.)	Multilevel logistic analysis	Region, family size, father's educational level, mother's educational level, brushing frequency, dental floss use, dental visit.	*
							≤1/>1 time per week		2.11 (1.80, 2.49)			
2	El Tantawi, et al. [32] (2018)	Male student	685	13-15y	GI (≥2)	Daily use of sugary drinks	Yes (vs No)	Beta	0.35 (-0.06, 0.75)	Linear regression analysis	Mother's and father's education, type of residence, number of households, plaque index score, brushing frequency, smoking status	-
						Daily use of sugary foods	Yes (vs No)		-0.07 (-0.45, 0.32)			-
3	Fann, et al. [27] (2016)	General population	10,022	35-44y	CPI (≥3)	Soft drinks including carbonated beverage, cola, milk tea, and juice or asparagus juice	≤2 times/week	OR	1.00 (Ref.)	Logistic regression analysis	Age, sex, educational level, cigarette smoking, regular teeth-brushing, BMI, hyperglycemia, WBC, intake of fruits	*
							3–4 times/week		1.05 (0.92, 1.20)			
							≥5 times/week		1.17 (1.03, 1.34)			
					LA (≥ 1mm)		≤2 times/week		1.00 (Ref.)			*
							3–4 times/week		1.17 (1.03, 1.32)			
							≥5 times/week		1.14 (1.01, 1.29)			
4	Jagahashi, et al. [33] (2012)	Students	504	6-12y	GI (≥2)	Sugars containing food and beverage (chocolate, jam, cake, biscuits, muffins)	≤3 time/day	OR	1.00 (Ref.)	Logistic regression analysis	Oral hygiene, tooth brushing	*
							>3 time/day		1.82 (1.07, 3.09)			
5	Kyaw, et al. [28] (2020)	Students	537	Mean: 10.6y (1SD=0.7)	PMA index	Sweet snack	Daily (vs not daily)	Beta	-0.12 (-1.28, 1.04)	Linear regression analysis	Sex, parents' occupation, tooth brushing frequency, mouth-rinsing habits, dental visits, OHI-S score, bacteria level	-
						Sweet drink	Daily (vs not daily)		1.65 (0.54, 2.76)			*
6	Lula, et al. [17] (2014)	General population	2,437	18-25y	PPD≥3mm and BOP	Added sugar intake (food and beverage) including cakes, cookies, brownies, ice cream, ice milk, milkshakes, chocolate candy, fudge, Hi-C, Tang, Kool-Aid, cola, and soda	0 time/month	PR	1.00 (Ref.)	Poisson regression analysis	Sex, age, race-ethnicity, education, poverty-income ratio, self-reported diabetes, serum cotinine concentration, refined starchy food intake, BMI	*
							1-22 times/month		1.52 (1.15, 2.01)			
							23-396 times/month		1.54 (1.10, 2.14)			

7	Menezes, et al. [30] (2019)	Pregnant women in 22nd to 25th week of pregnancy	1,185	N/A	PPD≥4mm and BOP	Soft drinks	0 time/week	MR	1.00 (Ref.)	Zero-inflated Poisson regression	Maternal age, family income, pre-pregnancy obesity, diastolic blood pressure	*
							1-5 times/week		1.08 (0.90, 1.30)			
							6-35 times/week		1.34 (1.03, 1.75)			
					CAL≥4mm		0 time/week		1.00 (Ref.)			-
							1-5 times/week		1.05 (0.95, 1.16)			
							6-35 times/week		0.95 (0.82, 1.12)			
8	Moreira, et al. [31] (2021)	General population	2,515	18-19y	BOP, PPD≥4mm, CAL≥4mm	Added sugar intake was estimated as the percentage of daily calories from added sugar present in beverages as soft drink, fruit-flavored juice, chocolate drinks, energy drinks, and a wide range of food groups, such as dairy products, bread, cookies, breakfast cereals, desserts, chocolate, mayonnaise, salty snacks, and cold cuts	<10% of total energy intake	MR	1.00 (Ref.)	Zero-inflated Poisson regression analysis	Household income, adolescent educational level, sex, smoking status, alcohol use	*
							≥10% of total energy intake		1.15 (1.03, 1.29)			*
9	Simon, et al. [36] (2003)	Students	1,736	12-18y	BOP and calculus	Sweetened drink (e.g. milk, tea, soft drinks)	≤2.5 times/week	OR	1.00 (Ref.)	Logistic regression analysis	Staple food, teeth cleaning	-
							2.6-5.0 times/week		0.93 (0.69, 1.26)			
							>5 times/week		0.92 (0.59, 1.42)			
						Sweets (e.g. Chocolate, cake, candy, cookies, and ice cream)	≤4 times/week		1.00 (Ref.)			*
							5-10 times/week		1.04 (0.76, 1.44)			
							>10 times/week		1.86 (1.18, 2.94)			
10	Song, et al. [16] (2016)	General population	5,517	19-39y	CPI≥3	Carbonated beverages	No	OR	1.00 (Ref.)	Logistic regression analysis	Age, sex, BMI, smoking status, drinking habits, exercise, metabolic syndrome, frequency of tooth brushing, use of secondary oral products, dental checkup, consumption of coffee	*
							≤1 time/month		1.11 (0.80, 1.53)			
							≤1 times/week		1.40 (0.98, 2.02)			
							≥2 times/week		1.47 (1.06, 2.03)			
11	Vilarrasa, et al. [34] (2021)	Patients with dental implants	169	mean 54.5y (1SD=11.7)	PPD, BOP, suppuration, radiographic bone level	Regular sugar consumption	No	OR	1.00 (Ref.)	Multinomial logistic regression	Sex, oral dryness, history of periodontitis and SPT compliance, no. of caries	-
							Yes		3.24 (0.95, 11.12)			
							No		1.00 (Ref.)			*
							Yes		5.38 (1.39, 20.87)			

12	Vitosyte, et al. [35] (2022)	General population	453	35-74	Number of teeth with PPD≥ 4mm)	Frequency of eating or drinking any of following food/drink: cake, sweet buns/breads, jam, honey, sweets, candies, soft, drinks, tea with sugar, coffee with sugar.	Lower<br>Moderate<br>Higher	Beta	0.00 (Ref.)<br>-0.56 (-1.83, 0.63)<br>-0.87 (-2.36, 0.62)	Linear regression analysis	Smoking frequency, alcohol use, dental visit, use of fluoride toothpaste, tooth-brushing frequency	-
**Cohort study**											
13	Yoshihara, et al. [29] (2009)	Independen t older adults	261	70y	No of teeth with periodontal event (≥ 3mm deeper PPD from baseline)	Cereals, nuts and seeds, sugar and sweeteners, confectioneries	g/kg	Beta	0.19 (0.01, 0.38)	Linear regression analysis	Dark green and yellow vegetables intake, alcohol (g/kg), number of remaining teeth at baseline.	*

NOTE: [a] * indicates the significance association between free sugar intake and periodontal diseases (p<0.05).

Abbreviation: PD=periodontal diseases; BOP=bleeding on probing; GI=Löe and Sillness's gingival index; CPI=community periodontal index; LA=loss of attachment; PPD=probing pocket depth; CAL=clinical attachment level; OR=odds ratio; PR=prevalence ratio; MR=mean ratio; 95%CI=95% confidence interval.

Supplementary Table S2. Funding source and conflicts of interests of the included studies.

ID	First author (publication year)	Country	Targeted population	Participants (n)	Age at baseline	funding source	conflicts of interests
**Cross-sectional study**							
1	Chen, et al. [26] (2020)	China	Adolescents without serious health problem	4,525	12y	National Natural Science Foundation of China grant (No. 81970948), and Scientific Research in the Public Interest grant (No.201502002).	No
2	El Tantawi, et al. [32] (2018)	Saudi Arabia	Male student	685	13-15y	None	No
3	Fann, et al. [27] (2016)	Taiwan	General population	10,022	35-44y	None	No
4	Jagahashi, et al. [33] (2012)	Syrian Arab Republic	Students	504	6-12y	N/A	N/A
5	Kyaw, et al. [28] (2020)	Myanmar	Students	537	Mean: 10.6y (1SD=0.7)	Dental hygiene research grant 2018 from the FUTOKU Foundation (Tokyo, Japan), and the Tokyo Medical and Dental University (Tokyo, Japan).	No
6	Lula, et al. [17] (2014)	US	General population	2,437	18-25y	Fundação de Amparo à Pesquisa e ao Desenvolvimento Científico e Tecnológico do Maranhão (FAPEMA)	No
7	Menezes, et al. [30] (2019)	Brazil	Pregnant women in 22nd to 25th week of pregnancy	1,185	N/A	CNPq (Conselho Nacional de Desenvolvimento Científico e Tecnológico—Portuguese acronym for the Brazilian National Research Council), grants 471923/2011-7 and 561058/2010-5; FAPESP (Fundação de Amparo à Pesquisa do Estado de São Paulo—Portuguese acronym for the São Paulo Research Foundation), grant 2008-53593-0; and FAPEMA (Fundação de Amparo à Pesquisa e ao Desenvolvimento Científico e Tecnológico do Maranhão—Portuguese acronym for the Maranhão State Research Foundantion), grants 0035/2008, 00356/11, and 01362-11.	No
8	Moreira, et al. [31] (2021)	Brazil	General population	2,515	18-19y	FAPEMA (Maranhão State Foundation for Research and Scientific and Technological Development), CNPq (National Council for Scientific and Technological Development) and CAPES (Coordination for the Improvement of Higher Education Personnel).	No
9	Simon, et al. [36] (2003)	Ethiopia	Students in public and private school	1,736	12-18y	N/A	N/A
10	Song, et al. [16] (2016)	South Korea	General population	5,517	19-39y	N/A	N/A
11	Vilarrasa, et al. [34] (2021)	Spain	Patients with dental implants	169	mean 54.5y (1SD=11.7)	None	No
12	Vitosyte, et al. [35] (2022)	Lithuania	General population	453	35-74	The Lithuanian National Oral Health Survey was funded by the Borrow Foundation, United Kingdom, grant number 3303900.	No
**Cohort study**							
13	Yoshihara, et al. [29] (2009)	Japan	Independent older adults	261	70y	Grant-in-Aid from the Ministry of Health and Welfare of Japan (H16-Iryo-001), by the Ministry of Education, Science, Sports and Culture of Japan (Grant No. 17592177), and by Japan Daily Association (18–06).	N/A
